# Examining the association between genetic liability for schizophrenia and psychotic symptoms in Alzheimer’s disease

**DOI:** 10.1038/s41398-019-0592-5

**Published:** 2019-10-22

**Authors:** Byron Creese, Evangelos Vassos, Sverre Bergh, Lavinia Athanasiu, Iskandar Johar, Arvid Rongve, Ingrid Tøndel Medbøen, Miguel Vasconcelos Da Silva, Eivind Aakhus, Fred Andersen, Francesco Bettella, Anne Braekhus, Srdjan Djurovic, Giulia Paroni, Petroula Proitsi, Ingvild Saltvedt, Davide Seripa, Eystein Stordal, Tormod Fladby, Dag Aarsland, Ole A. Andreassen, Clive Ballard, Geir Selbaek, on behalf of the AddNeuroMed consortium and the Alzheimer’s Disease Neuroimaging Initiative

**Affiliations:** 1 University of Exeter Medical School, Exeter, UK; 2 Norwegian, Exeter and King’s College Consortium for Genetics of Neuropsychiatric Symptoms in Dementia, Exeter, UK; 3 Social Genetic and Developmental Psychiatry Centre, Institute of Psychiatry, Psychology and Neuroscience, King’s College London, London, UK; 4 Research Centre of Age-Related Functional Decline and Disease, Innlandet Hospital Trust, Pb 68, 2312 Ottestad, Norway; 5 Norwegian National Advisory Unit on Ageing and Health, Vestfold Hospital Trust, Tønsberg, Norway; 6 NORMENT, Institute of Clinical Medicine, University of Oslo, Oslo, Norway; 7 NORMENT, Division of Mental Health and Addiction, Oslo University Hospital, Oslo, Norway; 8 Department of Old Age Psychiatry, Institute of Psychiatry, Psychology and Neuroscience, King’s College London, London, UK; 9 Department of Research and Innovation, Helse Fonna, Haugesund, Norway; 10 Department of Clinical Medicine, University of Bergen, Bergen, Norway; 11 Department of Geriatric Medicine, Oslo University Hospital, Oslo, Norway; 12 Department of Community Medicine, University of Tromsø, Tromsø, Norway; 13 Department of Neurology, Oslo University Hospital, Oslo, Norway; 14 NORMENT, Department of Clinical Science, University of Bergen, Bergen, Norway; 15 Department of Medical Genetics, Oslo University Hospital, Oslo, Norway; 16 Complex Structure of Geriatrics, Department of Medical Sciences, Fondazione IRCCS “Casa Sollievo della Sofferenza”, San Giovanni Rotondo, FG Italy; 17 Department of Basic and Clinical Neuroscience, Institute of Psychiatry, Psychology and Neuroscience, King’s College London, London, UK; 18 Geriatric Department, St. Olav Hospital, University Hospital of Trondheim, Trondheim, Norway; 19 Department of Neuromedicine and Movement Science, Norwegian University of Science and Technology, Trondheim, Norway; 20 Department of Mental Health, Norwegian University of Science and Technology, Trondheim, Norway; 21 Department of Psychiatry, Namsos Hospital, Namsos, Norway; 22 Department of Neurology, Akershus University Hospital, Lørenskog, Norway; 23 Institute of Clinical Medicine, Campus Ahus, University of Oslo, Oslo, Norway; 24 Centre for Age-Related Medicine, Stavanger University Hospital, Stavanger, Norway; 25 Faculty of Medicine, University of Oslo, Oslo, Norway

**Keywords:** Genomics, Schizophrenia

## Abstract

Psychosis (delusions or hallucinations) in Alzheimer’s disease (AD + P) occurs in up to 50% of individuals and is associated with significantly worse clinical outcomes. Atypical antipsychotics, first developed for schizophrenia, are commonly used in AD + P, suggesting shared mechanisms. Despite this implication, little empirical research has been conducted to examine whether there are mechanistic similarities between AD + P and schizophrenia. In this study, we tested whether polygenic risk score (PRS) for schizophrenia was associated with AD + P. Schizophrenia PRS was calculated using Psychiatric Genomics Consortium data at ten GWAS *p* value thresholds (*P*_T_) in 3111 AD cases from 11 cohort studies characterized for psychosis using validated, standardized tools. Association between PRS and AD + P status was tested by logistic regression in each cohort individually and the results meta-analyzed. The schizophrenia PRS was associated with AD + P at an optimum *P*_T_ of 0.01. The strongest association was for delusions where a one standard deviation increase in PRS was associated with a 1.18-fold increased risk (95% CI: 1.06–1.3; *p* = 0.001). These new findings point towards psychosis in AD—and particularly delusions—sharing some genetic liability with schizophrenia and support a transdiagnostic view of psychotic symptoms across the lifespan.

## Introduction

Psychosis in Alzheimer’s disease (AD + P)—broadly comprising delusions and hallucinations—is experienced by up to 50% of people over the course of the illness, with prevalence peaking in the later stages^1^. AD + P is associated with accelerated cognitive decline (independent of disease duration), higher mortality rates and distress to both people with the disease and their carers^2–4^. Moreover, there are wider societal implications with long-term follow-up studies indicating that AD + P is associated with a shorter time to nursing home care^5^. Despite these compelling reasons for effective management, there is a critical treatment gap, with no licensed treatments available in many jurisdictions. Atypical antipsychotics—developed first for schizophrenia—are frequently used to treat AD + P (in many countries off label) and, while they have some modest benefits, are associated with considerable harms, including a 1.5- to 1.8-fold increase in mortality and a threefold increase in stroke^6^.

Clinically useful alternatives to antipsychotics are scarce. There are only two new antipsychotic compounds in phase II or later stages of development (pimavanserin and MP-101) but both are refinements of existing mechanisms of action of atypical antipsychotics targeting mechanisms relevant to schizophrenia (e.g. 5HT2A, mGluR2/3) and side effects remain a concern^7^. The limited understanding of the biological mechanisms underpinning AD + P represents a major challenge to the effective targeting of existing treatments and the identification of novel treatment targets.

One key question is whether some or all of the psychotic symptoms experienced by people with AD have a similar basis to schizophrenia. Phenomenologically the psychotic symptoms in each are different; in AD visual hallucinations are more common than auditory hallucinations, delusions are usually simple, and the first rank symptoms of schizophrenia are very rare. In addition, schizophrenia is characterized by both positive and negative symptoms. While negative symptoms can also accompany psychosis in AD, consensus is yet to be reached on whether these other neuropsychiatric symptoms form part of the AD + P clinical syndrome. Despite the different phenomenology, atypical antipsychotics confer some treatment benefits in some cases of AD + P^8^, and similar neuropsychological deficits in processing speed and executive function have been observed in individuals with very-late-onset schizophrenia-like psychosis and AD + P^9^, suggesting some overlap.

A transdiagnostic hypothesis, proposing a mechanistic overlap between AD + P and schizophrenia, is gaining some traction^10^ and is supported by genetic studies of psychosis in adolescence, the general adult population and Huntington’s disease all showing overlap with schizophrenia^11–13^. In view of these findings and the high heritability of schizophrenia^14^ and of AD + P (estimated at 81% and 61% respectively)^15^, it is logical to look for common genetic underpinnings of the two disorders. Comparative studies examining common mechanisms between AD and schizophrenia point towards synaptic elimination and disruption, and telomere length^16–18^, but studies examining AD + P specifically and schizophrenia are less common. It is of note that a recent major GWAS reported a nominally significant genetic correlation between schizophrenia and AD^19^. It is possible that the presence of psychosis in the AD sample (which was unknown in this study) contributed to part of the association, underscoring the need for dissection of the AD phenotype by psychosis status. In a small study, a copy number variant (CNV) with significant overlap of a duplicated region implicated in schizophrenia and autism (16p11.2) was found in two of 440 AD + P cases but not in AD without psychosis, or in those with more occasional symptoms^20^. Linkage studies have also implicated regions of the genome in AD + P that have been identified in schizophrenia^21,22^. Another approach is to examine whether polygenic risk for schizophrenia, summarized in a score (the weighted sum of risk associated alleles) with better discrimination properties than single markers^23^, is associated with AD + P. Work in this area is limited to only one recent study which, surprisingly, reported that a genetic risk score comprising 94 SNPs reaching genome-wide significance for association with schizophrenia was lower in AD + P compared with AD without psychosis^24^. While this study represents an important preliminary step in AD + P research, a full genome-wide polygenic risk score (PRS) approach is imperative to address this key question^25,26^.

Another largely unexplored avenue in AD + P genetic research relates to the split of delusions and hallucinations. Although the two symptoms frequently co-occur in AD, there is evidence from longitudinal cohort studies indicating that 10–20% of people experience hallucinations without delusions and that the two symptoms are associated with different clinical outcomes^2,27^, suggesting the presence of two distinct clinical phenotypes. While it is commonplace to separate out composite psychotic symptoms in neuroimaging studies of AD + P^28,29^, their separate genetic associations have not yet been examined in any large-scale AD studies leveraging GWAS data^30^. This is a particularly relevant issue when assessing genetic overlap with schizophrenia where the emerging evidence from neuroimaging and the clinical similarity supports the hypothesis that shared etiology would be specific to delusions.

We conducted an analysis of the relationship between genetic liability for schizophrenia and AD + P with two principal objectives; firstly, we tested whether PRS for schizophrenia was associated with AD + P and secondly, we examined the association between the PRS and AD  with delusions.

## Methods

Ethical approval for this analysis protocol was obtained from University of Exeter Medical School Research Ethics Committee (Nov17/D/143).

### Cohorts

AD + P target data consisted of 3111 AD cases from 11 cohort studies in Europe and the USA: AddNeuroMed^31^ (Europe, longitudinal: assessment every 3 months for maximum 1 year), Alzheimer’s Disease Neuroimaging Initiative^32^ (ADNI; USA, longitudinal: assessment at baseline, 6, 12, 24 and 36 months for maximum 3 years), Istituto di Ricovero e Cura a Carattere Scientifico (IRCCS 1; Italy, cross-sectional), Health and Memory Study in Nord-Trøndelag^33^ (HMS; Norway, cross-sectional), Resource Use and Disease Couse in Dementia^34^ (REDIC; Norway, longitudinal: assessment every 6 months for maximum 2.5 years), Norwegian registry of persons assessed for cognitive symptoms^35^ (NorCog; Norway, cross-sectional), Samhandling mellom avdeling for alderspsykiatri og kommunale sykehjem (SAM-AKS; Norway, cross-sectional), The Dementia Study in Northern Norway^36^ (NordNorge, Norway, longitudinal: assessment at baseline and 1 year), Progression of Alzheimer’s Disease and Resource Use^37^ (PADR; Norway, longitudinal: assessment at baseline and 1 year), The Dementia Study in Western Norway^38^ (DemVest; Norway, longitudinal: assessment every 12 months maximum 6 years); and data from the National Alzheimer’s Coordinating Center (NACC; USA, longitudinal: assessment approximately every 12 months) and the National Institute on Aging Genetics Data Storage Site (NIAGADS), Table 1). Full cohort details are contained in the supplementary material and the Norwegian cohorts are also described in the latest GWAS of Alzheimer’s disease^19^. Informed consent was obtained by each study for all participants.

**Table 1:** Baseline characteristics by cohort

	*N*	Age				Gender		MMSE				Scale	Follow-up (years)^a,b^	Number of assessments done^b^	Array
		AD − P		AD + P		AD − P	AD + P	AD − P		AD + P					
		Mean	SD	Mean	SD	% male	% male	Mean	SD	Mean	SD				
AddNeuroMed	225	76	7	78	5.6	42	24	21	4.6	20	4.8	NPI	1	5	Illumina 610
ADNI	248	76	7.2	74	7.4	63	43	24	2.5	23	2.5	NPI-Q	3	4	Illumina OmniExpress
DemVest	80	77	8.3	76	5.5	23	38	24	2.4	23	2.4	NPI	5	6	Illumina OmniExpress
IRCCS 1	326	78	7.4	79	6.4	44	36	14	6.1	10	6.3	NPI	0	1	Illumina GSA
HMS	178	86	6.2	86	7.6	24	28	14	6.8	12	6.0	NPI	0	1	Illumina OmniExpress
NorCog	563	74	9.1	77	8.2	43	39	22	4.2	21	4.6	NPI-Q	0	1	Illumina OmniExpress
NordNorge	133	80	6.7	83	6.2	42	36	24	4.3	22	4.5	NPI	1	2	Illumina OmniExpress
PADR	106	76	6.6	77	6.6	35	30	21	4.3	21	4.4	NPI-Q	1	2	Illumina OmniExpress
REDIC	323	86	6.9	84	7.4	35	32	17	6.4	16	6.5	NPI	2	5	Illumina OmniExpress
SAM-AKS	93	86	6.8	86	5	29	38	16	5.0	15	5.2	NPI	0	1	Illumina OmniExpress
NACC	836	79	7.8	78	9	54	44	20	7.1	19	7.0	NPI-Q	2	3	Illumina 660/Omni Express
Total	3111	79	8.7	80	8.2	44	37	20	6	18	6.8	—	—	—	—

*NPI* Neuropsychiatric Inventory (full version), *NPI-Q* Neuropsychiatric Inventory—Questionnaire, *MMSE* Mini Mental State Examination

^a^‘0’ denotes that the study was cross-sectional (i.e. one assessment available)

^b^Figures are median

Some data used in the preparation of this article were obtained from the Alzheimer’s Disease Neuroimaging Initiative (ADNI) database (adni.loni.usc.edu). The ADNI was launched in 2003 as a public−private partnership, led by Principal Investigator Michael W. Weiner, MD. The primary goal of ADNI has been to test whether serial magnetic resonance imaging (MRI), positron emission tomography (PET), other biological markers, and clinical and neuropsychological assessment can be combined to measure the progression of mild cognitive impairment (MCI) and early Alzheimer’s disease (AD). For up-to-date information, see www.adni-info.org.

### AD clinical assessments

Diagnosis of AD was performed according to ICD-10 etiological diagnosis, NINCDS-ADRDA criteria or clinical diagnosis by psychiatrist or geriatrician. Longitudinal data were available for seven cohorts (ADNI, AddNeuroMed, DemVest, NordNorge, PADR, REDIC, NACC) and psychotic symptom classification was based on the maximum amount of follow-up data available. Any cases with a history of bipolar disorder or schizophrenia were excluded. For NorCog, PADR, REDIC, SAM-AKS, NACC and ADNI the necessary information on psychiatric history was extracted from source study data resulting in 3, 1, 2, 1, 31 and 1 exclusions, respectively. For AddNeuroMed, DemVest, IRCCS 1 and NordNorge this was an exclusion criterion applied at entry to those individual studies. No information about psychiatric history was available for the HMS study. Dementia severity was assessed in all cohorts by Mini Mental State Examination (MMSE) and psychotic symptoms were assessed by the Neuropsychiatric Inventory (NPI) or its short version, the Neuropsychiatric Inventory Questionnaire (NPI-Q), they are among the most widely used validated instruments to assess psychosis^39^. Psychotic symptoms are rated on the basis of items A (delusions) and B (hallucinations) of the NPI and NPI-Q. These are two different versions of the same scale, which are strongly correlated and have good between-rater and test−retest reliability, particularly for the psychosis items^31,40^. Ratings were carried about by trained research staff in all cohorts. In the full NPI, neuropsychiatric symptoms are coded as present or absent first. If rated present they are further scored according to their frequency (1–4) and severity (1–3) with the resulting scores multiplied to give an overall rating (i.e. possible scores are 1, 2, 3, 4, 6, 8, 9 and 12 with 0 indicating no symptoms). The NPI-Q is rated only on a scale of 0−3 according to the severity of the symptom. Both scales have been designed to be completed by verbal interview with a proxy informant who knows the person with AD well. Several diagnostic criteria for AD + P have been proposed but none have been adopted clinically, meaning that where in other psychiatric disorders medical records can be screened, in AD + P this would be unreliable and ratings on specific validated assessment scales must be used. Using such scales, we thus undertook examination of three related but progressively more homogenous psychotic phenotypes:

1. *Psychosis wide:* Psychosis present: the presence of delusions or hallucinations (NPI/NPI-Q item A or B > 0) at any point; No psychosis: no evidence of delusions or hallucinations (NPI/NPI-Q item A or B = 0) at any point in follow up.
2. *Psychosis narrow:* Psychosis present: the presence of delusions or hallucinations (NPI/NPI-Q item A or B > 0) at any point; No psychosis: here, an additional level of screening was applied to those rated as having no delusions or hallucinations. In these cases, if an individual was psychosis-free based on criteria for psychosis wide but had not yet reached a moderately severe dementia stage based on available data (defined as MMSE < 20) they were excluded from the analysis. This is a similar approach to that used in most previous AD + P genetic research^24,41^.
3. *Delusions narrow:* Delusions present: the presence of delusions (NPI/NPI-Q item A > 0) at any point during follow-up. Thus, the delusion group was the psychosis narrow group above with any individuals rated as having hallucinations only removed. No delusions: as per psychosis narrow.

### Genotyping and QC

The genotyping chips used are detailed in Table 1. Raw genotype data for individual cohorts underwent appropriate QC steps (implemented in PLINK). SNPs with a minor allele frequency ≤5% and a Hardy Weinberg equilibrium *p* < 10^−5^ were excluded. The SNP and individual genotype failure threshold was set at 5% and individuals with mean heterozygosity ±3 standard deviations were excluded. The analysis was restricted to individuals of European ancestry using genetic principal components computed by EIGENSTRAT. Related (pi-hat > 0.2) or duplicate individuals both within and between cohorts were excluded. Phasing (EAGLE2) and imputation (PBWT) was done via the Sanger Imputation Service using the Haplotype Reference Consortium (r1.1) reference panel on all cohorts. After imputation only SNPs with an imputation quality (INFO) score > 0.4 and MAF > 0.05 were retained. This resulted in 4,895,913 SNPs common across all 11 cohorts available to compute polygenic risk scores.

The most recently published schizophrenia GWAS data from the Psychiatric Genomics Consortium (PGC) was used as base data to generate PRS in the target AD sample^26^. SNPs with MAF < 0.1, INFO < 0.9 and indels were excluded from the base dataset to leave only the most informative SNPs and only one SNP from the extended MHC region was included^42^. As a positive control and to evaluate the specificity of the association we then generated PRS of height and depression using the latest GIANT consortium and PGC GWAS results^40,43^.

### Analysis

PRS for schizophrenia were generated in PRSice^44^ at the following ten GWAS *p* value thresholds (*P*_T_): 5 × 10^−8^, 1 × 10^−5^, 1 × 10^−4^, 1 × 10^−3^, 0.01, 0.05, 0.1, 0.2, 0.5 and 1. Clumping was performed (250 kb, r2 > 0.1) to retain only the SNP with the strongest association in each window. The resulting PRS were standardized (centering by mean, scaling by standard deviation) for the analysis.

Power was calculated using AVENGEME^45^, with schizophrenia parameters as set out in Palla and Dudbridge^45^, number of markers genotyped in both datasets was 76,213 (see section “Schizophrenia PRS is associated with AD psychosis status”), a prevalence of 40%^1^ and of 36%^1^ was used for psychosis and delusions, and case-control sample fractions as per Table 2. There are no data available for estimated covariance between AD + P and schizophrenia but if this value is assumed to be 0.08 (less than the 0.13 and 0.17 for schizophrenia and major depressive disorder and bipolar disorder estimated by AVENGEME^45^), this study has ≥80% power for each *P*_T_ ≥ 0.01 for psychosis and delusions respectively but <80% power below this value. All statistical analysis was implemented in R. For each cohort ten logistic regression models (one per *P*_T_) were run with each of the previously defined psychosis phenotypes as the binary outcome and the first ten ancestry principal components included as covariates. Disease severity is accounted for in our “narrow” phenotype definitions and as there is no strong evidence that age and gender are associated with AD + P^1^ so these were not included as covariates. Logistic regression assumptions were confirmed using the R “car” package. Proportion of variance explained (*R*^2^) by PRS, on the observed scale, was determined by subtracting the Nagelkerke’s pseudo-*R*^2^ of the null model from that of the full model. Regression coefficients for each *P*_T_ across all cohorts were then included in random effects meta-analyses to account for between-study variation in data collection protocols, frequency of psychosis and dementia severity^46–48^. Meta-analysis was undertaken using the “rma” function in the “metafor” package using the REML method^49^. Because the PRS calculated were correlated, a Bonferroni correction for multiple testing was considered too stringent. Using a correlation matrix of the ten PRS and the matSpD tool (https://gump.qimr.edu.au/general/daleN/matSpD/), the effective number of independent tests was determined to be 5 and the experiment-wide significance threshold for type I error rate of 5% determined to be *p* = 0.01. All tests reported are two-sided.

**Table 2:** Frequencies of symptoms by cohort for the three psychosis phenotypes

	Psychosis wide					Psychosis narrow					Delusions narrow				
	*N*	Absent		Present		*N*	Absent		Present		*N*	Absent		Present	
		*n*	%	*n*	%		*n*	%	*n*	%		*n*	%	*n*	%
AddNeuroMed	225	133	59	92	41	157	65	41	92	59	142	65	46	77	54
ADNI	248	183	74	65	26	117	52	44	65	56	99	52	53	47	47
DemVest	80	30	38	50	63	75	25	33	50	67	69	25	36	44	64
IRCCS 1	326	222	68	104	32	293	189	65	104	35	271	189	70	82	30
HMS	178	107	60	71	40	162	91	56	71	44	152	91	60	61	40
NorCog	563	402	71	161	29	288	127	44	161	56	260	127	49	133	51
NordNorge	133	105	79	28	21	45	17	38	28	62	38	17	45	21	55
PADR	106	62	58	44	42	83	39	47	44	53	80	39	49	41	51
REDIC	323	158	49	165	51	276	111	40	165	60	265	111	42	154	58
SAM-AKS	93	73	78	20	22	80	60	75	20	25	75	60	80	15	20
NACC	836	520	62	316	38	656	340	52	316	48	601	340	57	261	43
Total	3111	1995	64	1116	36	2232	1116	50	1116	50	2052	1116	54	936	46

Percentages may not sum to 100 due to rounding

## Results

On average across all 11 cohorts, individuals were in the mild-moderate stages of dementia at first assessment (mean MMSE of 19). Mean MMSE by cohort ranged from an MMSE of 12 (IRCCS 1) to 24 (ADNI) and this was a correlate of the prevalence of psychosis in each cohort (note the denominator would be the overall cohort *N* in Table 1), with cohorts that contained individuals with more severe dementia typically having a higher proportion of people with psychosis. Between cohorts, mean age at baseline ranged from 75 to 87 years and the proportion of male participants ranged from 26 to 59%. There was little difference in age between the psychosis and no psychosis groups across all studies but gender distributions did differ.

Frequency of the three psychosis phenotypes by cohort is shown in Table 2. Of the 3111 individuals screened, 1116 (36%) had psychosis (wide definition group). Of the 1995 who were rated as having no psychosis based on their assessment scale result alone, 879 had not yet reached the moderate stages of disease and so were excluded; 1116 AD + P cases and 1116 AD no psychosis “controls” were included in the analysis of the narrow phenotype of psychosis. In all, 936 cases met the criteria for having delusions narrow.

### Schizophrenia PRS is associated with AD psychosis status

**Fig. 1:**
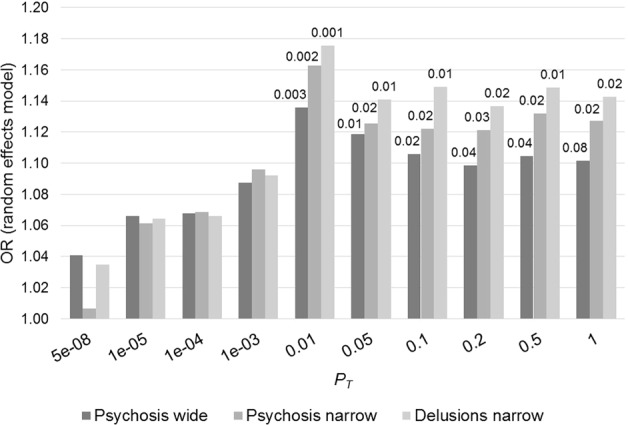
Odds ratios from random effects meta-analysis of AD psychosis wide, narrow and delusions narrow association with schizophrenia PRS. Each bar represents PRS composed of markers at ten different schizophrenia GWAS *P* value thresholds (*P*_T_). *P* values shown above each bar

After clumping, 76,213 independent variants were available for computing PRS. Random effects meta-analysis across the 11 cohorts showed the largest OR for the schizophrenia PRS at *P*_T_ = 0.01 and this was significantly associated with symptom status across the psychosis wide, psychosis narrow and delusions narrow phenotypes despite the progressively smaller sample size in each of these groups (OR: 1.14, 95% CI: 1.05–1.23, *p* = 0.003; OR: 1.16, 95% CI: 1.06–1.28, *p* = 0.004; OR: 1.18, 95% CI:1.06–1.30, *p* = 0.001 respectively) (see Fig. 1 and Table 3). PRS was also significantly associated with both the psychosis narrow and delusions narrow phenotypes at every *P*_T_ > 0.01. The largest effect size was observed in the delusions narrow group. Overall, there was no evidence of significant heterogeneity; *I*^2^ statistics were close to 0% for *P*_T_ = 0.01 across the three phenotypes.

**Table 3:** Random effects meta-analysis results for association between schizophrenia PRS across ten GWAS thresholds (*P*_T_) and AD + P

*P*_T_	nSNPs	Psychosis wide			Psychosis narrow			Delusions narrow		
		OR	95% CI	*p*	OR	95% CI	*p*	OR	95% CI	*p*
5 × 10^−08^	125	1.04	0.96–1.13	0.32	1.01	0.92–1.10	0.89	1.03	0.94–1.14	0.48
1 × 10^−05^	511	1.07	0.98–1.16	0.15	1.06	0.97–1.16	0.20	1.06	0.97–1.17	0.20
1 × 10^−04^	1147	1.07	0.96–1.18	0.21	1.07	0.96–1.19	0.21	1.07	0.96–1.18	0.21
1 × 10^−03^	2922	1.09	0.98–1.21	0.11	1.10	0.98–1.22	0.10	1.09	0.98–1.21	0.10
0.01	8709	1.14	1.05–1.23	0.003	1.16	1.06–1.28	0.002	1.18	1.06–1.30	0.001
0.05	19,656	1.12	1.03–1.22	0.01	1.13	1.02–1.24	0.02	1.14	1.03–1.26	0.01
0.1	28,143	1.11	1.01–1.21	0.02	1.12	1.02–1.24	0.02	1.15	1.04–1.28	0.01
0.2	40,253	1.10	1.01–1.20	0.04	1.12	1.01–1.24	0.03	1.14	1.02–1.26	0.02
0.5	61,727	1.10	1.00–1.22	0.04	1.13	1.02–1.25	0.02	1.15	1.03–1.28	0.01
1	76,213	1.10	0.99–1.23	0.08	1.13	1.02–1.25	0.02	1.14	1.03–1.27	0.02

*OR* odds ratio, odds ratio estimates may differ slightly from those represented in Fig. 1 due to rounding

**Fig. 2:**
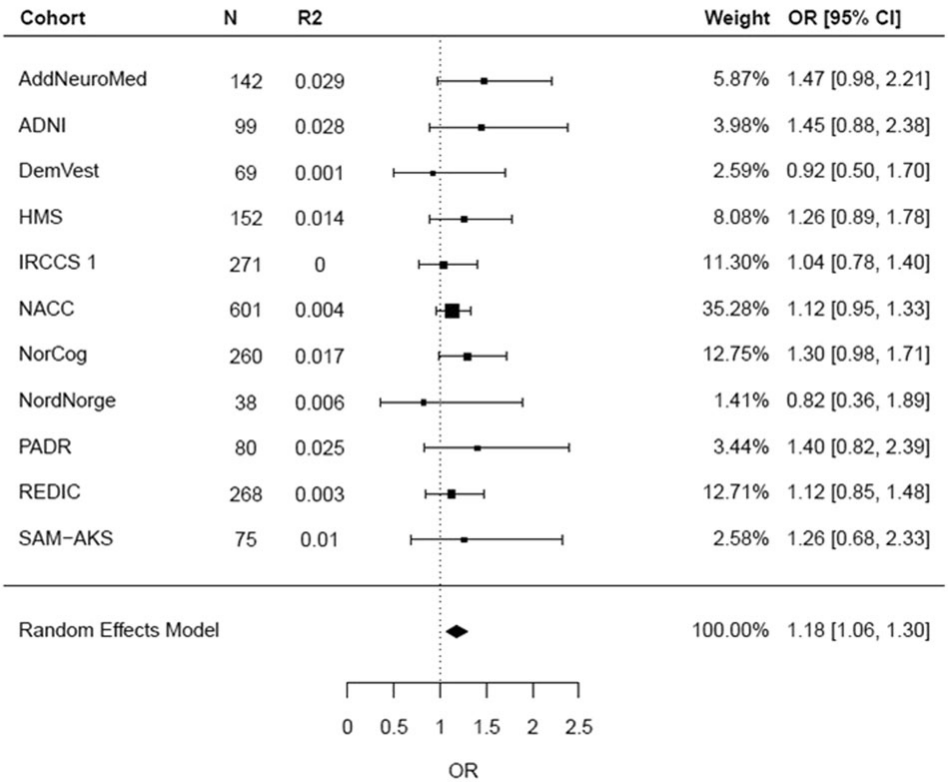
Forest plot of meta-analysis of delusions narrow for PRS calculated at *P*_T_ = 0.01 (i.e. 8709 SNPs). Overall estimate from random effects model is represented by the diamond below the individual study estimates

In the individual cohort analysis, we observed that the effect estimates of association between schizophrenia PRS and AD + P in nine of the 11 studies were in the same direction (OR > 1), albeit not statistically significantly (Supplementary Table 1). A forest plot of individual study estimates for delusions narrow at *P*_T_ = 0.01, the strongest association found in the above meta-analysis, is shown in Fig. 2. A similar plot at *P*_T_ = 1 for comparison is shown in the Supplementary material along with plots for psychosis wide and psychosis narrow phenotypes. The highest Nagelkerke’s *R*^2^ estimate was 2.9% (AddNeuroMed) and the lowest was <0.1% (IRCCS 1). An overall variance explained (Nagelkerke’s *R*^2^) in AD + P by schizophrenia PRS of 0.08% was estimated by calculating the weighted average *R*^2^ across the 11 studies. To determine the specificity of the signal, PRS for major depression (using the PGC GWAS^43^) and height (GIANT consortium GWAS^40^) were generated post-hoc at *P*_T_ *=* 1 and tested for association with delusions using the same procedure as described in the section “Analysis”. Neither PRS showed any evidence of association (major depression: OR: 1.03, 95% CI: 0.91–1.18, *p* = 0.61; height: OR: 0.99, 95% CI: 0.85–1.17, *p* = 0.99).

## Discussion

We set out to examine whether genetic risk for psychotic symptoms in AD (AD + P) is attributable to common schizophrenia variants. Using polygenic scoring, we found that schizophrenia PRS was associated with AD + P in a collection of over 3000 well-characterized cases and the association persisted as the AD + P phenotype was more precisely defined, despite the progressively smaller *N*. The largest effect size was observed at *P*_T_ = 0.01 which was associated with a 1.14-, 1.16- and 1.18-fold (per standard deviation increase in PRS) increased risk of psychosis (wide), psychosis (narrow) and delusions (narrow), respectively. In the individual cohort analysis, the odds ratios of 9 of the 11 studies were in the same direction (OR > 1). In all, these new findings suggest that AD + P is part of a spectrum of neuropsychiatric conditions characterized by psychosis across the lifespan. However, in common with other studies in psychiatric genomics PRS are yet not appropriate for symptom or disease course prediction in AD + P. Although the variance explained by schizophrenia PRS in AD + P is only modest, with the *R*^2^ estimates being less than 1%, this should be seen in the context of the same PRS explaining around 2.5% of the variance in bipolar disorder and 1% in MDD in a cross-disorder analysis of the Psychiatric Genomics Consortium with significantly larger target sample sizes^50^.

In line with our findings, a recent study in UK Biobank found psychotic experiences in the general population to be associated with PRS for schizophrenia, with the strongest association observed for delusions^12^. Several possible conclusions can be drawn from the finding that the association was still observed in the delusions phenotype in this study, despite a considerably smaller *N* compared with the broader psychosis phenotypes. This finding may point towards a subset of AD + P patients that have a more schizophrenia-like phenotype. More work is needed to investigate whether further diagnostic refinements to AD + P syndrome definitions are necessary, which may provide a more robust approach for pharmacological intervention trials. Related to this, from a methodological point of view, we show that there is a need for future studies in AD to consider delusions and hallucinations separately. We cannot rule out a genetic association between hallucinations in AD and schizophrenia in these cohorts but the evidence at present suggests a weaker association than for delusions. One might speculate that this is due to visual hallucinations in AD being more often the result of a broader range of causes (e.g. due to medication or delirium) than delusions, thus introducing more noise into the phenotype. The final wider implication is related to the schizophrenia PRS being associated with a broad spectrum of psychotic disorders and personality traits^11–13,50–52^. Our findings support a transdiagnostic explanation of delusions, which reaches into neurodegenerative disease and is underpinned by a degree of common genetic liability.

A key strength of our study is the detailed phenotyping with longitudinal data being available in 7 of the 11 cohorts. Rather than relying on medical record screens, which would be highly unreliable for AD + P given the lack of universally accepted and used diagnostic criteria, every individual in our analysis was assessed using specific, reliable assessment tools. We then used this data to dissect AD + P phenotype by focusing on delusions as well as the broader syndrome. We also followed previous research by taking extra measures to screen the “control” groups. This removed any cases in the mild stages of disease who had not yet developed symptoms (i.e. those still at risk^1^). This approach has been used in most previous genetic research but our extension to focus on delusions in AD + P is novel. Our finding that the association persisted with this more precision definition is consistent with genetic studies of other polygenic traits, like depression^43^.

For one study (HMS) data on history of major psychiatric conditions were not available. It is possible that some individuals with schizophrenia were present in this cohort; however, HMS is a cohort with a mean age of 87 so it is highly unlikely that the number would be more than one or two out of 178 people in the HMS cohort (this is also supported by the very small numbers we found among the other studies we screened). With over 3000 samples, this is, to our knowledge, the largest analysis of AD + P to exploit GWAS data^41^. We acknowledge that using different cohorts has led to some variability due to sampling but it is important to acknowledge that there are no single cohorts which are large enough to conduct an analysis of this kind and because of potential sampling and protocol variations across the individual studies we ensured an appropriate analysis was implemented to account for this variability; the same approach as used in other studies examining PRS in complex phenotypes^46–48^. We had access to raw individual-level clinical and genotype data, allowing us to run the same regression models in each study. This included undertaking the same QC across cohorts, imputing all chip data to the same reference panel and analyzing only SNPs present across all cohorts. After ensuring this standardized process was followed for each cohort we ran a random effects meta-analysis, allowing for the effect of the PRS on AD + P to vary across studies. In all, and in the absence of a single large enough study, these measures provide the most robust estimates, as reflected in the low heterogeneity statistics of the meta-analysis and the narrow range of effect estimates and overlapping confidence intervals across the 11 studies included (Fig. 2 and Supplementary Figs. 1–3). Finally, as with all similar studies, these results are not generalizable to individuals with non-European ancestry; there is an equal imperative to extend the genomics of AD + P to other populations as in AD itself.

A previous study that examined a genetic risk score at a more conservative *P*_T_ comprised of only 94 genome-wide significant schizophrenia SNPs found it to be lower in AD + P cases^24^. Our study is a similar size to this previous study, and the NACC data were used in both. Given that a PRS with only 94 SNPs will be a less powerful predictor than a full genome-wide score, it is possible larger studies will be needed to confirm associations at this more conservative *P*_T_. Nevertheless, schizophrenia is highly polygenic; tens of thousands of markers explain only 7% of the variance on the liability scale, while for optimum cross-trait case-control (e.g. schizophrenia and bipolar) prediction many thousands more SNPs are required^50^. In addition, cases of schizophrenia in the PGC study (used as base sample to estimate PRS) include patients with both a positive and negative syndrome. There is evidence that negative and disorganized symptoms are more heritable than positive, which—although we report a positive association—may reduce the power of schizophrenia PRS at more conservative *P*_T_ to discriminate AD cases with or without psychotic symptoms^53,54^. Accordingly, a full account of association between schizophrenia and AD + P should exploit the full polygenic nature of schizophrenia; our study is the first to do this and the findings represent an important further step towards a complete account of the relationship between common schizophrenia variants and AD + P. Another important milestone will be an appropriately powered discovery GWAS of AD + P and all of these points underscore the need for increasing samples sizes in this field.

In summary, these findings support shared genetic liability between schizophrenia and psychosis in AD. This provides a strong rationale for further work to build a clearer clinical and biological understanding of the psychosis syndrome in AD, an urgently needed step for better management and treatment development.

## Supplementary information

Supplemental Material

Supplemental Material
