# Supplementary material for: Examining the association between genetic liability for schizophrenia and psychotic symptoms in Alzheimer’s disease
Source: Transl Psychiatry. 2019 Oct 22;9:273. doi: 10.1038/s41398-019-0592-5 (PMC6805870; doi:10.1038/s41398-019-0592-5)

**Cohorts**

**DemVest**

Participants recruited when having a consultation for cognitive decline at five outpatient clinics in geriatric medicine, neurology and geriatric psychiatry in two counties in western Norway. The inclusion period was from March 2005 to March 2007.

Participants with a diagnosis of dementia according to the Diagnostic and Statistical Manual of Mental Disorders, Fourth Edition (DSM-IV), AD was diagnosed according to the criteria of the National Institute of Neurological and Communicative Disorders and Stroke/Alzheimer’s

Disease and Related Disorders Association (NINCDS-ADRDA). Participants had to have a Mini-Mental State Examination (MMSE) score of 20 at baseline to be included in the study. Exclusion criteria were acute delirium, terminal illness, previous severe mental disorder, or major physical disease.

The participants were followed up with assessments every 12 months until death, and a subgroup underwent neuropathological examination post mortem [1, 2].

**NorCog**

The Norwegian registry of persons assessed for cognitive symptoms (NorCog) is a national quality- and research registry collecting extensive clinical data and biobank material at outpatient clinics across Norway. Patients from NorCog included in the current study were recruited from memory clinics, geriatric-, and old-age psychiatric outpatient clinics in three of the four regional health authorities responsible for specialist healthcare in Norway. Participants underwent a comprehensive clinical assessment according to a standardized protocol including verbal interviews of the patient and proxy informant, neurocognitive testing, as well as a psychiatric, physical, and neurological examination. Diagnosis of AD was performed according to the ICD-10 criteria.

**PADR**

The Progression of Alzheimer’s Disease and Resource use (PADR) study is a longitudinal observational study with assessments at the time of diagnostic workup (baseline) and follow-up after a mean of 24 months (range 16–37, 80% between 20 and 28 months). The PADR study recruited patients from three memory clinics in Norway. Inclusion criteria were MCI or dementia at baseline, living at home, able to give informed consent, and have a proxy informant available. Exclusion criteria were: not fluent in Norwegian, severe physical illness and not living in close proximity to one of the memory clinics. Two of the memory clinics were affiliated to NorCog (described above), but PADR is an independent sample. The Regional Committee for Medical and Health Research Ethics in South East Norway approved the study (REC number 2011/531). Patients received oral and written information and gave written consent to participate. Only patients with capacity to consent were recruited at baseline, as recommended by REC.

The National Institute of Neurological and Communicative Disorders and Stroke-Alzheimer’s Disease and Related Disorders Association (NINCDS-ADRDA) were used to diagnose AD. Diagnoses were assigned by the study researchers reviewing all available data from the baseline examination; no diagnoses were revised based on the subsequent disease course. Decreased beta amyloid and/or elevated phosphor-tau or total-tau in CSF supported AD diagnosis.

**Nord-Norge**

AD patients were recruited from general practice and population screening based on the same diagnostic criteria, as described below, between January 2006 and December 2007. Inclusion criteria were individuals aged 65 years or over with a MMSE sum score between 10 and 30. Exclusion criteria were delirium and behavioral disturbances interfering with assessments, reluctance to participate, and inability to understand the purpose of the study, or relatives/caregivers disapproving participation. The study was registered as an International Standard Randomised Controlled Trial within ClinicalTrials.gov and approved by the following bodies; The Regional Committee for Medical Research Ethics in Northern Norway, The Privacy Ombudsman for Research, The Directory of Health and Social Welfare and The Norwegian Medicine Agency including the EudraCT database (no 2004-002613-37). Each participant gave a written informed consent co-signed by a spouse, a close relative or a guardian. Diagnosis of dementia was set by GPs and discussed with at least one specialist in geriatric medicine according to the ICD-10 criteria [21], AD according to the Statistical Manual of Mental Disorders fourth edition (DSM-IV-TR) and probable AD according to National Institute of Neurological Disorders and Stroke-Alzheimer Disease and Related Disorders' (NINCDS-ADRDA) criteria. Disagreement or uncertainty about the diagnostic subtypes regarding 12 patients was solved by consulting a third specialist in geriatric medicine.

**REDIC-NH**

In the Resource Use and Disease Couse in dementia - Nursing Home (REDIC-NH) cohort, participants were recruited at admission to one of 47 nursing homes in 4 counties in Norway. The inclusion was from March 2012 to November 2014. Patients eligible for inclusion in the study were 65 years or older, or younger than 65 years with established dementia, with an expected stay in the NH of more than four weeks. The only exclusion criterion was a life expectancy of less than six weeks.

The participants were assessed with the NPI-NH for NPS, in addition to several other assessment tools for depression, cognition, ADL function and physical disease.

Based on all available information, AD according to ICD-10 was independently diagnosed by two of the authors (G.S. and S.B) both of whom are experienced old age psychiatrists and researchers, with the possibility of consulting a third specialist, to reach a consensus.

Patients were followed up with biannual assessments until death.

**SAM-AKS**

The Samhandling mellom avdeling for alderspsykiatri og kommunale sykehjem (SAM-AKS; translation: Collaboration between the Department of Old Age Psychiatry and nursing homes in the municipality.) cohort was recruited from nursing homes in two counties in Norway, with the same in- and exclusion criteria, the assessment tools, the same diagnostic criteria and the same routine for a research diagnosis as the REDIC-NH cohort.

**HMS**

In the HMS cohort, participants were recruited from nursing homes in 24 municipalities in one county in middle Norway. Inclusion was between 2010 and 2011. Patients were eligible for inclusion if they had stayed in a nursing home for at least 14 days. Patients were diagnosed by the same method as the REDIC and SAM-AKS cohorts. A written consent was collected from the participants, or from their next of kin if the participants were unable to give informed consent. The Regional Committee for Medical and Health Research Ethics in South East Norway approved the study (REC number 2011/1738 A).

No follow-up assessments were performed.

**Alzheimer’s Disease Neuroimaging Initiative (ADNI)**

Data used in the preparation of this article were obtained from the Alzheimer’s Disease Neuroimaging Initiative (ADNI) database (adni.loni.usc.edu). The ADNI was launched in 2003 as a public-private partnership, led by Principal Investigator Michael W. Weiner, MD. The primary goal of ADNI has been to test whether serial magnetic resonance imaging (MRI), positron emission tomography (PET), other biological markers, and clinical and neuropsychological assessment can be combined to measure the progression of mild cognitive impairment (MCI) and early Alzheimer’s disease (AD). For up-to-date information, see [www.adni-info.org](http://www.adni-info.org). Diagnosis of AD was made according to the NINCDS-ADRDA.

**AddNeuroMed**

The AddNeuroMed (ANM) study involves six cross-European collection study sites; London (United Kingdom), Toulouse (France), Perugia (Italy), Kuopio (Finland), Lodz (Poland), and Thessaloniki (Greece) and has been described previously ([Lovestone, et al., 2009](#_ENREF_3)). Participants were assessed once every three months for 1 year (5 assessments in total). Diagnosis of AD was made according to the NINCDS-ADRDA.

**IRCCS 1**

All clinical records selected were part of a collection of clinical data and DNA samples recruited in a frame of a wider study investigating the role of genetic factors in frailty and disability of elderly patients attending a geriatric ward. The study was conducted according to the Declaration of Helsinki, the Guidelines for Good Clinical Practice and the guidelines for Strengthening the Reporting of Observational Studies in Epidemiology, and approved by the ethic committee of the IRCCS Casa Sollievo della Sofferenza (Prot. N. 3877/DS). Written informed consent for research was available from each patient. All subjects recruited in the study were Caucasian, not including people of Jewish, Eastern European, nor North African descent, with all individuals having Southern Italian ancestry and living in Southern Italy from at least three generations.

The medical record system of the IRCCS Casa Sollievo della Sofferenza were used to identify all diagnoses of AD (diagnosed according to NINCDS-ADRDA criteria) from January to December 2016. Diagnostic criteria included the assessment of the general medical status by means of a structured interview, a clinical evaluation and a review of records from the patients’ general practitioners, and the administration of the comprehensive geriatric assessment (CGA); and a deeper screening of the cognitive status with the Mini-Mental State Examination (MMSE) and the Clinical Dementia Rating Scale after a brief interview with the caregiver.

**National Alzheimer’s Coordinating Center (NACC)**

This analysis used data from 25 Alzheimer’s Disease Centers and the following Alzheimer’s Disease Center genotype datasets: NG00022, NG00023 and NG00024. Clinical data analyzed was based from assessments conducted between September 2005 and May 2017. This longitudinal data was collected using the Uniform Data Set (UDS; V1, V2, and V3 implemented in 2005, 2008 and 2015 respectively), using a prospective, standardized, and longitudinal clinical evaluation of the subjects in the National Institute on Aging's ADC Program. Diagnosis of AD was made according to NINCDS/ADRDA criteria (V1 and V2) or the NIA-AA criteria for AD (V3).

**Forest plots**

Supplementary figure 1: Forest plot of meta-analysis of delusions narrow for PRS calculated at PT=0.01 (i.e. 8,129 SNPs) and PT=1 (i.e. 76,244 SNPs).

**P_T_=0.01**

**P_T_=0.01**

**P_T_=1**

**P_T_=1**

Supplementary figure 2: Forest plot of meta-analysis of psychosis narrow for PRS calculated at PT=0.01 (i.e. 8,129 SNPs) and PT=1 (i.e. 76,244 SNPs).

Supplementary figure 3: Forest plot of meta-analysis of psychosis wide for PRS calculated at PT=0.01 (i.e. 8,129 SNPs) and PT=1 (i.e. 76,244 SNPs).

**P_T_=1**

**P_T_=0.01**


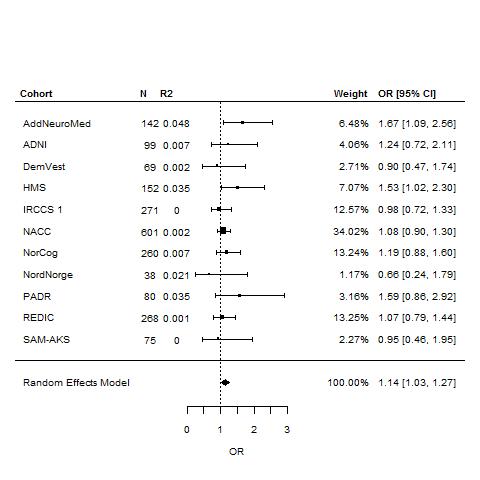


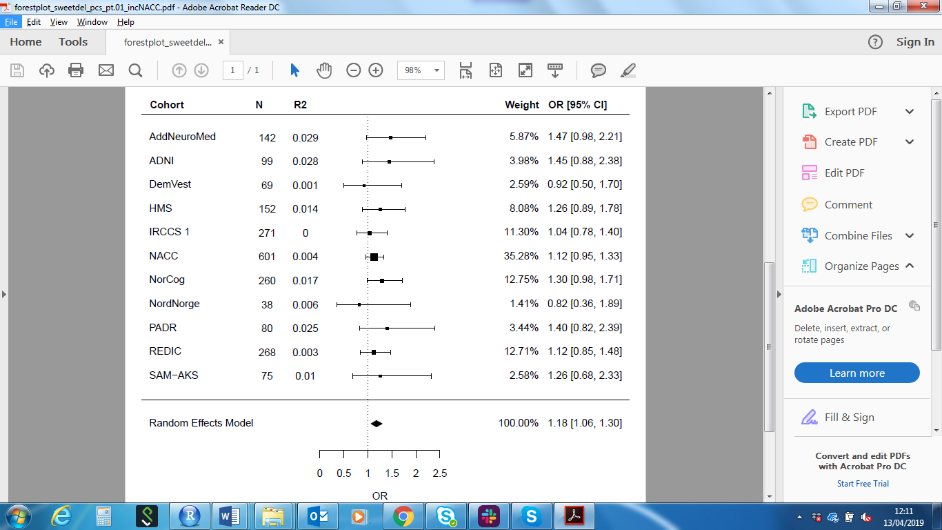


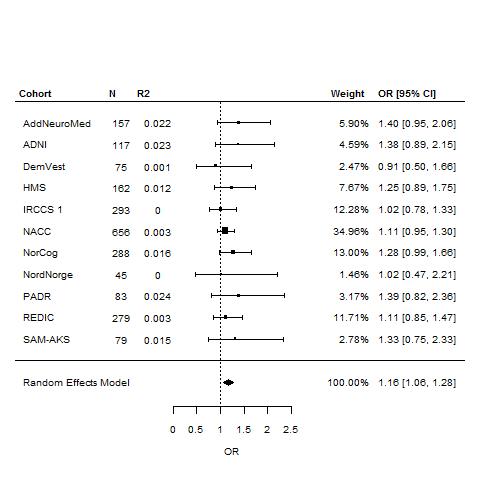


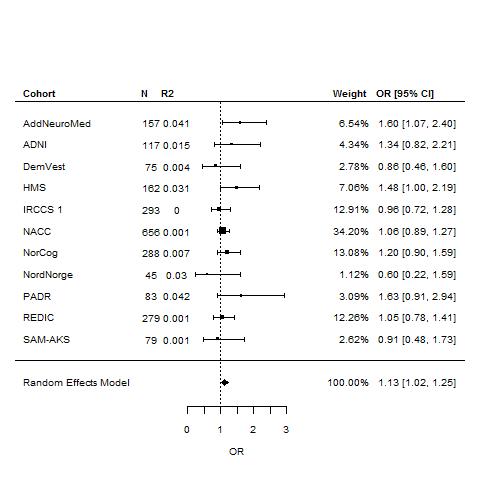


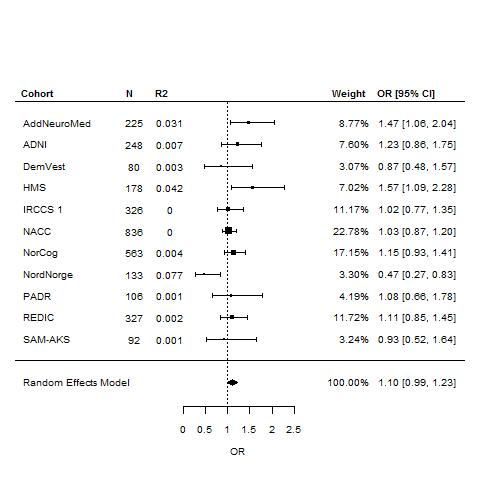

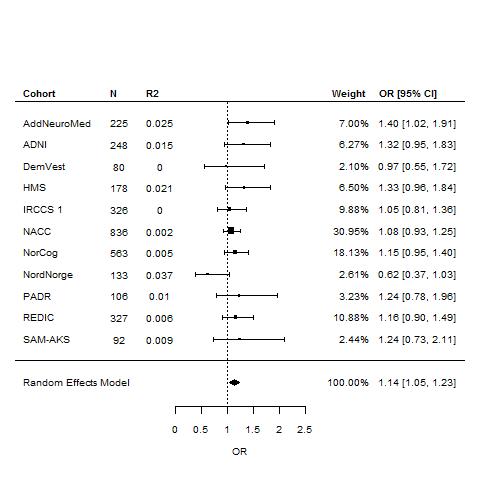

Supplement: Supplementary file 1 — Supplemental Material [file 41398_2019_592_MOESM1_ESM.docx]
