# Supplementary material for: Examining the association between genetic liability for schizophrenia and psychotic symptoms in Alzheimer’s disease
Source: Transl Psychiatry. 2019 Oct 22;9:273. doi: 10.1038/s41398-019-0592-5 (PMC6805870; doi:10.1038/s41398-019-0592-5)
Supplement: Supplementary file 2 — Supplemental Material [file 41398_2019_592_MOESM2_ESM.pdf]

Supplementary Table 1: Individual Cohort Regression Results

Pt	Estimate	Std. Error	z value	Pr(>|z|)	r2.out	Study	Analysis
5e.8	0.3209	0.1605	1.9997	0.0455	0.0229	ADNI	Psychosis wide
1e.5	0.2656	0.1741	1.5262	0.1269	0.0132	ADNI	Psychosis wide
1e.4	0.2627	0.1674	1.5694	0.1166	0.0140	ADNI	Psychosis wide
0.001	0.3276	0.1522	2.1529	0.0313	0.0267	ADNI	Psychosis wide
0.01	0.2751	0.1687	1.6303	0.1030	0.0153	ADNI	Psychosis wide
0.05	0.3387	0.1826	1.8547	0.0636	0.0197	ADNI	Psychosis wide
0.1	0.3268	0.1813	1.8021	0.0715	0.0186	ADNI	Psychosis wide
0.2	0.2960	0.1827	1.6205	0.1051	0.0150	ADNI	Psychosis wide
0.5	0.1997	0.1812	1.1019	0.2705	0.0069	ADNI	Psychosis wide
1	0.2033	0.1813	1.1213	0.2622	0.0071	ADNI	Psychosis wide
5e.8	0.0322	0.1550	0.2078	0.8354	0.0002	AddNeuroMed	Psychosis wide
1e.5	0.0541	0.1416	0.3823	0.7023	0.0008	AddNeuroMed	Psychosis wide
1e.4	0.0251	0.1440	0.1742	0.8617	0.0002	AddNeuroMed	Psychosis wide
0.001	0.2535	0.1569	1.6158	0.1061	0.0146	AddNeuroMed	Psychosis wide
0.01	0.3338	0.1597	2.0906	0.0366	0.0246	AddNeuroMed	Psychosis wide
0.05	0.2562	0.1575	1.6272	0.1037	0.0148	AddNeuroMed	Psychosis wide
0.1	0.3459	0.1635	2.1151	0.0344	0.0251	AddNeuroMed	Psychosis wide
0.2	0.3241	0.1623	1.9967	0.0459	0.0224	AddNeuroMed	Psychosis wide
0.5	0.3817	0.1653	2.3094	0.0209	0.0301	AddNeuroMed	Psychosis wide
1	0.3877	0.1662	2.3328	0.0197	0.0307	AddNeuroMed	Psychosis wide
5e.8	0.3359	0.3260	1.0302	0.3029	0.0153	DemVest	Psychosis wide
1e.5	0.0101	0.3065	0.0330	0.9737	0.0000	DemVest	Psychosis wide
1e.4	0.1285	0.3118	0.4122	0.6802	0.0024	DemVest	Psychosis wide
0.001	0.0207	0.2858	0.0726	0.9421	0.0001	DemVest	Psychosis wide
0.01	-0.0269	0.2912	-0.0923	0.9264	0.0001	DemVest	Psychosis wide
0.05	0.0549	0.2808	0.1956	0.8449	0.0005	DemVest	Psychosis wide
0.1	-0.0109	0.2835	-0.0385	0.9693	0.0000	DemVest	Psychosis wide
0.2	-0.0774	0.2908	-0.2662	0.7901	0.0010	DemVest	Psychosis wide
0.5	-0.1376	0.2969	-0.4635	0.6430	0.0030	DemVest	Psychosis wide
1	-0.1422	0.3011	-0.4723	0.6367	0.0031	DemVest	Psychosis wide
5e.8	0.0097	0.0718	0.1353	0.8924	0.0000	NACC	Psychosis wide
1e.5	-0.0601	0.0718	-0.8374	0.4024	0.0011	NACC	Psychosis wide
1e.4	-0.1030	0.0733	-1.4062	0.1597	0.0032	NACC	Psychosis wide
0.001	-0.0581	0.0717	-0.8103	0.4178	0.0011	NACC	Psychosis wide
0.01	0.0744	0.0759	0.9803	0.3269	0.0015	NACC	Psychosis wide
0.05	0.0643	0.0794	0.8107	0.4175	0.0011	NACC	Psychosis wide
0.1	0.0593	0.0815	0.7280	0.4666	0.0009	NACC	Psychosis wide
0.2	0.0457	0.0818	0.5585	0.5765	0.0005	NACC	Psychosis wide
0.5	0.0312	0.0816	0.3823	0.7023	0.0002	NACC	Psychosis wide
1	0.0258	0.0818	0.3149	0.7528	0.0002	NACC	Psychosis wide
5e.8	-0.0459	0.1648	-0.2783	0.7808	0.0006	HMS	Psychosis wide
1e.5	0.1494	0.1485	1.0057	0.3145	0.0073	HMS	Psychosis wide
1e.4	0.2216	0.1521	1.4564	0.1453	0.0153	HMS	Psychosis wide
0.001	0.1820	0.1542	1.1798	0.2381	0.0100	HMS	Psychosis wide
0.01	0.2827	0.1657	1.7066	0.0879	0.0213	HMS	Psychosis wide
0.05	0.3311	0.1687	1.9628	0.0497	0.0282	HMS	Psychosis wide
0.1	0.3416	0.1721	1.9851	0.0471	0.0288	HMS	Psychosis wide
0.2	0.4142	0.1880	2.2029	0.0276	0.0357	HMS	Psychosis wide
0.5	0.4404	0.1890	2.3301	0.0198	0.0400	HMS	Psychosis wide
1	0.4540	0.1900	2.3896	0.0169	0.0421	HMS	Psychosis wide
5e.8	0.0124	0.0948	0.1310	0.8958	0.0000	NorCog	Psychosis wide
1e.5	0.1418	0.0969	1.4639	0.1432	0.0054	NorCog	Psychosis wide
1e.4	0.1511	0.0980	1.5411	0.1233	0.0060	NorCog	Psychosis wide
0.001	0.0605	0.0968	0.6250	0.5319	0.0010	NorCog	Psychosis wide
0.01	0.1395	0.0992	1.4062	0.1597	0.0050	NorCog	Psychosis wide

0.05	0.1398	0.1009	1.3854	0.1659	0.0049	NorCog	Psychosis wide
0.1	0.1088	0.1001	1.0865	0.2773	0.0030	NorCog	Psychosis wide
0.2	0.1241	0.1011	1.2273	0.2197	0.0038	NorCog	Psychosis wide
0.5	0.1358	0.1044	1.3009	0.1933	0.0043	NorCog	Psychosis wide
1	0.1363	0.1049	1.2993	0.1938	0.0043	NorCog	Psychosis wide
5e.8	0.0281	0.2387	0.1178	0.9062	0.0001	NordNorge	Psychosis wide
1e.5	0.0043	0.2373	0.0182	0.9854	0.0000	NordNorge	Psychosis wide
1e.4	-0.1403	0.2437	-0.5757	0.5649	0.0036	NordNorge	Psychosis wide
0.001	-0.3368	0.2659	-1.2666	0.2053	0.0177	NordNorge	Psychosis wide
0.01	-0.4795	0.2616	-1.8330	0.0668	0.0370	NordNorge	Psychosis wide
0.05	-0.6286	0.2848	-2.2073	0.0273	0.0543	NordNorge	Psychosis wide
0.1	-0.5755	0.2744	-2.0971	0.0360	0.0488	NordNorge	Psychosis wide
0.2	-0.6279	0.2749	-2.2837	0.0224	0.0582	NordNorge	Psychosis wide
0.5	-0.7257	0.2904	-2.4987	0.0125	0.0712	NordNorge	Psychosis wide
1	-0.7512	0.2900	-2.5908	0.0096	0.0771	NordNorge	Psychosis wide
5e.8	0.1081	0.2029	0.5329	0.5941	0.0035	PADR	Psychosis wide
1e.5	0.1777	0.2236	0.7947	0.4268	0.0077	PADR	Psychosis wide
1e.4	-0.1941	0.2375	-0.8171	0.4139	0.0082	PADR	Psychosis wide
0.001	0.0258	0.2555	0.1010	0.9196	0.0001	PADR	Psychosis wide
0.01	0.2116	0.2352	0.8997	0.3683	0.0099	PADR	Psychosis wide
0.05	0.1272	0.2421	0.5253	0.5994	0.0034	PADR	Psychosis wide
0.1	0.0567	0.2390	0.2373	0.8124	0.0007	PADR	Psychosis wide
0.2	0.0212	0.2473	0.0858	0.9316	0.0001	PADR	Psychosis wide
0.5	0.0706	0.2542	0.2778	0.7812	0.0009	PADR	Psychosis wide
1	0.0767	0.2544	0.3013	0.7632	0.0011	PADR	Psychosis wide
5e.8	-0.0522	0.1238	-0.4217	0.6732	0.0007	REDIC	Psychosis wide
1e.5	0.0661	0.1242	0.5321	0.5947	0.0011	REDIC	Psychosis wide
1e.4	0.1855	0.1147	1.6174	0.1058	0.0107	REDIC	Psychosis wide
0.001	0.2286	0.1192	1.9175	0.0552	0.0150	REDIC	Psychosis wide
0.01	0.1492	0.1281	1.1653	0.2439	0.0055	REDIC	Psychosis wide
0.05	0.1564	0.1328	1.1780	0.2388	0.0056	REDIC	Psychosis wide
0.1	0.0830	0.1341	0.6190	0.5359	0.0016	REDIC	Psychosis wide
0.2	0.1027	0.1351	0.7600	0.4472	0.0023	REDIC	Psychosis wide
0.5	0.1129	0.1367	0.8258	0.4089	0.0028	REDIC	Psychosis wide
1	0.1032	0.1380	0.7478	0.4546	0.0023	REDIC	Psychosis wide
5e.8	0.2600	0.2722	0.9554	0.3394	0.0135	SAMAKS	Psychosis wide
1e.5	0.4894	0.3014	1.6239	0.1044	0.0397	SAMAKS	Psychosis wide
1e.4	0.4272	0.3064	1.3941	0.1633	0.0298	SAMAKS	Psychosis wide
0.001	0.2897	0.3023	0.9581	0.3380	0.0139	SAMAKS	Psychosis wide
0.01	0.2155	0.2706	0.7964	0.4258	0.0093	SAMAKS	Psychosis wide
0.05	0.0459	0.2884	0.1593	0.8734	0.0004	SAMAKS	Psychosis wide
0.1	-0.1435	0.2852	-0.5030	0.6149	0.0037	SAMAKS	Psychosis wide
0.2	-0.0936	0.2876	-0.3254	0.7449	0.0015	SAMAKS	Psychosis wide
0.5	-0.0911	0.2914	-0.3126	0.7546	0.0014	SAMAKS	Psychosis wide
1	-0.0769	0.2927	-0.2626	0.7929	0.0010	SAMAKS	Psychosis wide
5e.8	0.0437	0.1263	0.3461	0.7293	0.0005	IRCCS1	Psychosis wide
1e.5	-0.0119	0.1250	-0.0948	0.9244	0.0000	IRCCS1	Psychosis wide
1e.4	-0.0289	0.1202	-0.2405	0.8100	0.0002	IRCCS1	Psychosis wide
0.001	-0.0564	0.1257	-0.4484	0.6539	0.0008	IRCCS1	Psychosis wide
0.01	0.0466	0.1344	0.3466	0.7289	0.0005	IRCCS1	Psychosis wide
0.05	-0.0272	0.1357	-0.2002	0.8413	0.0002	IRCCS1	Psychosis wide
0.1	0.0322	0.1375	0.2345	0.8146	0.0002	IRCCS1	Psychosis wide
0.2	-0.0051	0.1401	-0.0362	0.9712	0.0000	IRCCS1	Psychosis wide
0.5	0.0480	0.1423	0.3374	0.7358	0.0005	IRCCS1	Psychosis wide
1	0.0210	0.1425	0.1475	0.8828	0.0001	IRCCS1	Psychosis wide
5e.8	0.1164	0.2066	0.5634	0.5732	0.0034	ADNI	Psychosis narrow
1e.5	0.1829	0.2372	0.7712	0.4406	0.0064	ADNI	Psychosis narrow
1e.4	0.2473	0.2260	1.0940	0.2740	0.0130	ADNI	Psychosis narrow

0.001	0.3674	0.2037	1.8037	0.0713	0.0366	ADNI	Psychosis narrow
0.01	0.3229	0.2247	1.4370	0.1507	0.0227	ADNI	Psychosis narrow
0.05	0.4492	0.2550	1.7617	0.0781	0.0342	ADNI	Psychosis narrow
0.1	0.5135	0.2599	1.9758	0.0482	0.0429	ADNI	Psychosis narrow
0.2	0.4487	0.2618	1.7137	0.0866	0.0323	ADNI	Psychosis narrow
0.5	0.2938	0.2565	1.1452	0.2521	0.0142	ADNI	Psychosis narrow
1	0.2956	0.2535	1.1663	0.2435	0.0147	ADNI	Psychosis narrow
5e.8	-0.0530	0.1991	-0.2659	0.7903	0.0005	AddNeuroMed	Psychosis narrow
1e.5	0.0050	0.1844	0.0274	0.9782	0.0000	AddNeuroMed	Psychosis narrow
1e.4	0.0796	0.1780	0.4474	0.6546	0.0015	AddNeuroMed	Psychosis narrow
0.001	0.2922	0.1915	1.5259	0.1270	0.0180	AddNeuroMed	Psychosis narrow
0.01	0.3362	0.1981	1.6970	0.0897	0.0223	AddNeuroMed	Psychosis narrow
0.05	0.2885	0.1894	1.5236	0.1276	0.0179	AddNeuroMed	Psychosis narrow
0.1	0.3634	0.1970	1.8451	0.0650	0.0264	AddNeuroMed	Psychosis narrow
0.2	0.4039	0.2005	2.0147	0.0439	0.0317	AddNeuroMed	Psychosis narrow
0.5	0.4588	0.2042	2.2469	0.0246	0.0398	AddNeuroMed	Psychosis narrow
1	0.4704	0.2065	2.2775	0.0228	0.0409	AddNeuroMed	Psychosis narrow
5e.8	0.2401	0.3390	0.7084	0.4787	0.0079	DemVest	Psychosis narrow
1e.5	0.0216	0.3412	0.0632	0.9496	0.0001	DemVest	Psychosis narrow
1e.4	0.1124	0.3329	0.3375	0.7358	0.0018	DemVest	Psychosis narrow
0.001	-0.0212	0.3021	-0.0703	0.9439	0.0001	DemVest	Psychosis narrow
0.01	-0.0946	0.3060	-0.3092	0.7572	0.0015	DemVest	Psychosis narrow
0.05	0.0025	0.2866	0.0086	0.9931	0.0000	DemVest	Psychosis narrow
0.1	-0.0375	0.2926	-0.1281	0.8980	0.0003	DemVest	Psychosis narrow
0.2	-0.0848	0.3055	-0.2774	0.7814	0.0012	DemVest	Psychosis narrow
0.5	-0.1375	0.3121	-0.4407	0.6594	0.0030	DemVest	Psychosis narrow
1	-0.1509	0.3165	-0.4766	0.6337	0.0035	DemVest	Psychosis narrow
5e.8	-0.0151	0.0786	-0.1925	0.8473	0.0001	NACC	Psychosis narrow
1e.5	-0.0266	0.0779	-0.3417	0.7326	0.0002	NACC	Psychosis narrow
1e.4	-0.0796	0.0797	-0.9990	0.3178	0.0020	NACC	Psychosis narrow
0.001	-0.0326	0.0768	-0.4241	0.6715	0.0004	NACC	Psychosis narrow
0.01	0.1037	0.0814	1.2738	0.2027	0.0032	NACC	Psychosis narrow
0.05	0.0761	0.0854	0.8910	0.3729	0.0016	NACC	Psychosis narrow
0.1	0.0872	0.0886	0.9848	0.3247	0.0019	NACC	Psychosis narrow
0.2	0.0729	0.0900	0.8100	0.4179	0.0013	NACC	Psychosis narrow
0.5	0.0639	0.0901	0.7092	0.4782	0.0010	NACC	Psychosis narrow
1	0.0613	0.0903	0.6785	0.4975	0.0009	NACC	Psychosis narrow
5e.8	-0.1270	0.1739	-0.7306	0.4650	0.0042	HMS	Psychosis narrow
1e.5	0.0391	0.1564	0.2501	0.8025	0.0005	HMS	Psychosis narrow
1e.4	0.1271	0.1602	0.7936	0.4274	0.0049	HMS	Psychosis narrow
0.001	0.0458	0.1674	0.2739	0.7842	0.0006	HMS	Psychosis narrow
0.01	0.2193	0.1738	1.2620	0.2070	0.0125	HMS	Psychosis narrow
0.05	0.2740	0.1753	1.5625	0.1182	0.0192	HMS	Psychosis narrow
0.1	0.2872	0.1793	1.6014	0.1093	0.0202	HMS	Psychosis narrow
0.2	0.3520	0.1957	1.7989	0.0720	0.0255	HMS	Psychosis narrow
0.5	0.3787	0.1979	1.9132	0.0557	0.0289	HMS	Psychosis narrow
1	0.3929	0.1987	1.9767	0.0481	0.0309	HMS	Psychosis narrow
5e.8	0.0326	0.1230	0.2653	0.7908	0.0003	NorCog	Psychosis narrow
1e.5	0.2352	0.1347	1.7460	0.0808	0.0139	NorCog	Psychosis narrow
1e.4	0.1870	0.1303	1.4355	0.1511	0.0094	NorCog	Psychosis narrow
0.001	0.1874	0.1291	1.4511	0.1467	0.0096	NorCog	Psychosis narrow
0.01	0.2479	0.1335	1.8577	0.0632	0.0158	NorCog	Psychosis narrow
0.05	0.1242	0.1355	0.9169	0.3592	0.0038	NorCog	Psychosis narrow
0.1	0.0960	0.1356	0.7083	0.4788	0.0023	NorCog	Psychosis narrow
0.2	0.1209	0.1371	0.8817	0.3779	0.0035	NorCog	Psychosis narrow
0.5	0.1807	0.1456	1.2410	0.2146	0.0070	NorCog	Psychosis narrow
1	0.1788	0.1460	1.2244	0.2208	0.0068	NorCog	Psychosis narrow
5e.8	0.5185	0.3841	1.3499	0.1771	0.0505	NordNorge	Psychosis narrow

1e.5	0.5159	0.3685	1.3999	0.1616	0.0546	NordNorge	Psychosis narrow
1e.4	0.6511	0.4342	1.4995	0.1338	0.0644	NordNorge	Psychosis narrow
0.001	0.1790	0.4440	0.4031	0.6868	0.0043	NordNorge	Psychosis narrow
0.01	0.0151	0.3979	0.0378	0.9698	0.0000	NordNorge	Psychosis narrow
0.05	-0.2551	0.4957	-0.5145	0.6069	0.0070	NordNorge	Psychosis narrow
0.1	-0.3874	0.5050	-0.7670	0.4431	0.0159	NordNorge	Psychosis narrow
0.2	-0.3948	0.4919	-0.8026	0.4222	0.0175	NordNorge	Psychosis narrow
0.5	-0.4487	0.4989	-0.8994	0.3685	0.0223	NordNorge	Psychosis narrow
1	-0.5148	0.4997	-1.0303	0.3029	0.0296	NordNorge	Psychosis narrow
5e.8	0.0888	0.2290	0.3878	0.6982	0.0023	PADR	Psychosis narrow
1e.5	0.2444	0.2459	0.9939	0.3203	0.0153	PADR	Psychosis narrow
1e.4	-0.1247	0.2683	-0.4650	0.6419	0.0033	PADR	Psychosis narrow
0.001	0.0377	0.2852	0.1323	0.8947	0.0003	PADR	Psychosis narrow
0.01	0.3310	0.2702	1.2250	0.2206	0.0236	PADR	Psychosis narrow
0.05	0.3932	0.2770	1.4196	0.1557	0.0320	PADR	Psychosis narrow
0.1	0.4027	0.2762	1.4583	0.1447	0.0337	PADR	Psychosis narrow
0.2	0.4126	0.2863	1.4411	0.1495	0.0328	PADR	Psychosis narrow
0.5	0.5008	0.3027	1.6546	0.0980	0.0433	PADR	Psychosis narrow
1	0.4895	0.3002	1.6302	0.1031	0.0420	PADR	Psychosis narrow
5e.8	-0.1044	0.1382	-0.7554	0.4500	0.0027	REDIC	Psychosis narrow
1e.5	0.0524	0.1371	0.3819	0.7025	0.0007	REDIC	Psychosis narrow
1e.4	0.1510	0.1256	1.2019	0.2294	0.0069	REDIC	Psychosis narrow
0.001	0.1929	0.1320	1.4612	0.1440	0.0103	REDIC	Psychosis narrow
0.01	0.1075	0.1406	0.7644	0.4446	0.0028	REDIC	Psychosis narrow
0.05	0.1292	0.1460	0.8851	0.3761	0.0038	REDIC	Psychosis narrow
0.1	0.0470	0.1460	0.3218	0.7476	0.0005	REDIC	Psychosis narrow
0.2	0.0744	0.1481	0.5027	0.6152	0.0012	REDIC	Psychosis narrow
0.5	0.0605	0.1491	0.4059	0.6848	0.0008	REDIC	Psychosis narrow
1	0.0490	0.1508	0.3250	0.7452	0.0005	REDIC	Psychosis narrow
5e.8	0.1557	0.2701	0.5766	0.5642	0.0053	SAMAKS	Psychosis narrow
1e.5	0.4370	0.3007	1.4532	0.1462	0.0344	SAMAKS	Psychosis narrow
1e.4	0.3674	0.3221	1.1407	0.2540	0.0213	SAMAKS	Psychosis narrow
0.001	0.3677	0.3442	1.0681	0.2855	0.0189	SAMAKS	Psychosis narrow
0.01	0.2818	0.2885	0.9770	0.3286	0.0153	SAMAKS	Psychosis narrow
0.05	0.0264	0.3170	0.0833	0.9336	0.0001	SAMAKS	Psychosis narrow
0.1	-0.1691	0.3113	-0.5430	0.5871	0.0047	SAMAKS	Psychosis narrow
0.2	-0.1251	0.3194	-0.3915	0.6954	0.0024	SAMAKS	Psychosis narrow
0.5	-0.1079	0.3225	-0.3346	0.7379	0.0018	SAMAKS	Psychosis narrow
1	-0.0896	0.3266	-0.2743	0.7838	0.0012	SAMAKS	Psychosis narrow
5e.8	0.0361	0.1295	0.2786	0.7805	0.0004	IRCCS1	Psychosis narrow
1e.5	-0.0278	0.1304	-0.2132	0.8311	0.0002	IRCCS1	Psychosis narrow
1e.4	-0.0431	0.1226	-0.3516	0.7251	0.0006	IRCCS1	Psychosis narrow
0.001	-0.0865	0.1286	-0.6722	0.5014	0.0020	IRCCS1	Psychosis narrow
0.01	0.0165	0.1373	0.1204	0.9042	0.0001	IRCCS1	Psychosis narrow
0.05	-0.0725	0.1400	-0.5177	0.6046	0.0012	IRCCS1	Psychosis narrow
0.1	-0.0193	0.1399	-0.1380	0.8902	0.0001	IRCCS1	Psychosis narrow
0.2	-0.0643	0.1437	-0.4477	0.6544	0.0009	IRCCS1	Psychosis narrow
0.5	-0.0148	0.1463	-0.1014	0.9192	0.0000	IRCCS1	Psychosis narrow
1	-0.0378	0.1470	-0.2570	0.7972	0.0003	IRCCS1	Psychosis narrow
5e.8	0.0964	0.2181	0.4418	0.6586	0.0024	ADNI	Delusions narrow
1e.5	0.2197	0.2719	0.8079	0.4191	0.0082	ADNI	Delusions narrow
1e.4	0.2991	0.2521	1.1866	0.2354	0.0180	ADNI	Delusions narrow
0.001	0.3693	0.2284	1.6168	0.1059	0.0344	ADNI	Delusions narrow
0.01	0.3715	0.2533	1.4669	0.1424	0.0278	ADNI	Delusions narrow
0.05	0.4488	0.2781	1.6138	0.1066	0.0334	ADNI	Delusions narrow
0.1	0.4998	0.2855	1.7506	0.0800	0.0395	ADNI	Delusions narrow
0.2	0.3301	0.2763	1.1947	0.2322	0.0181	ADNI	Delusions narrow
0.5	0.2049	0.2765	0.7409	0.4587	0.0069	ADNI	Delusions narrow

1	0.2112	0.2743	0.7699	0.4413	0.0074	ADNI	Delusions narrow
5e.8	0.0072	0.2119	0.0340	0.9729	0.0000	AddNeuroMed	Delusions narrow
1e.5	0.0904	0.1988	0.4548	0.6493	0.0017	AddNeuroMed	Delusions narrow
1e.4	0.1261	0.1842	0.6846	0.4936	0.0039	AddNeuroMed	Delusions narrow
0.001	0.3361	0.1972	1.7049	0.0882	0.0243	AddNeuroMed	Delusions narrow
0.01	0.3857	0.2084	1.8509	0.0642	0.0287	AddNeuroMed	Delusions narrow
0.05	0.3187	0.2030	1.5697	0.1165	0.0205	AddNeuroMed	Delusions narrow
0.1	0.4257	0.2099	2.0283	0.0425	0.0346	AddNeuroMed	Delusions narrow
0.2	0.4695	0.2127	2.2077	0.0273	0.0413	AddNeuroMed	Delusions narrow
0.5	0.5017	0.2147	2.3365	0.0195	0.0467	AddNeuroMed	Delusions narrow
1	0.5131	0.2172	2.3622	0.0182	0.0478	AddNeuroMed	Delusions narrow
5e.8	0.3625	0.3636	0.9969	0.3188	0.0171	DemVest	Delusions narrow
1e.5	0.0659	0.3447	0.1913	0.8483	0.0006	DemVest	Delusions narrow
1e.4	0.1133	0.3483	0.3253	0.7450	0.0018	DemVest	Delusions narrow
0.001	-0.0301	0.3149	-0.0956	0.9239	0.0002	DemVest	Delusions narrow
0.01	-0.0843	0.3141	-0.2684	0.7884	0.0012	DemVest	Delusions narrow
0.05	0.0584	0.3078	0.1898	0.8495	0.0006	DemVest	Delusions narrow
0.1	0.0260	0.3169	0.0820	0.9346	0.0001	DemVest	Delusions narrow
0.2	-0.0148	0.3260	-0.0454	0.9638	0.0000	DemVest	Delusions narrow
0.5	-0.0856	0.3300	-0.2595	0.7953	0.0011	DemVest	Delusions narrow
1	-0.1028	0.3355	-0.3063	0.7593	0.0016	DemVest	Delusions narrow
5e.8	0.0186	0.0825	0.2256	0.8215	0.0001	NACC	Delusions narrow
1e.5	-0.0335	0.0814	-0.4121	0.6803	0.0004	NACC	Delusions narrow
1e.4	-0.0688	0.0836	-0.8227	0.4107	0.0015	NACC	Delusions narrow
0.001	-0.0214	0.0802	-0.2673	0.7893	0.0002	NACC	Delusions narrow
0.01	0.1170	0.0850	1.3765	0.1687	0.0042	NACC	Delusions narrow
0.05	0.0933	0.0891	1.0471	0.2951	0.0024	NACC	Delusions narrow
0.1	0.1136	0.0930	1.2214	0.2219	0.0033	NACC	Delusions narrow
0.2	0.0896	0.0944	0.9494	0.3424	0.0020	NACC	Delusions narrow
0.5	0.0821	0.0945	0.8687	0.3850	0.0017	NACC	Delusions narrow
1	0.0796	0.0948	0.8394	0.4013	0.0015	NACC	Delusions narrow
5e.8	-0.0737	0.1808	-0.4078	0.6834	0.0014	HMS	Delusions narrow
1e.5	0.1012	0.1630	0.6204	0.5350	0.0032	HMS	Delusions narrow
1e.4	0.1158	0.1643	0.7047	0.4810	0.0041	HMS	Delusions narrow
0.001	0.0444	0.1712	0.2591	0.7955	0.0006	HMS	Delusions narrow
0.01	0.2286	0.1777	1.2867	0.1982	0.0138	HMS	Delusions narrow
0.05	0.3126	0.1813	1.7240	0.0847	0.0250	HMS	Delusions narrow
0.1	0.3456	0.1876	1.8422	0.0654	0.0286	HMS	Delusions narrow
0.2	0.4063	0.2049	1.9831	0.0474	0.0333	HMS	Delusions narrow
0.5	0.4195	0.2076	2.0206	0.0433	0.0345	HMS	Delusions narrow
1	0.4241	0.2079	2.0399	0.0414	0.0351	HMS	Delusions narrow
5e.8	0.0192	0.1274	0.1510	0.8800	0.0001	NorCog	Delusions narrow
1e.5	0.1811	0.1412	1.2826	0.1996	0.0083	NorCog	Delusions narrow
1e.4	0.1678	0.1358	1.2359	0.2165	0.0077	NorCog	Delusions narrow
0.001	0.1872	0.1368	1.3687	0.1711	0.0094	NorCog	Delusions narrow
0.01	0.2603	0.1414	1.8408	0.0657	0.0172	NorCog	Delusions narrow
0.05	0.1195	0.1407	0.8492	0.3958	0.0036	NorCog	Delusions narrow
0.1	0.1032	0.1420	0.7268	0.4673	0.0026	NorCog	Delusions narrow
0.2	0.1149	0.1424	0.8069	0.4197	0.0032	NorCog	Delusions narrow
0.5	0.1796	0.1513	1.1866	0.2354	0.0070	NorCog	Delusions narrow
1	0.1745	0.1519	1.1487	0.2507	0.0066	NorCog	Delusions narrow
5e.8	0.5032	0.4203	1.1972	0.2312	0.0457	NordNorge	Delusions narrow
1e.5	0.3546	0.3683	0.9627	0.3357	0.0292	NordNorge	Delusions narrow
1e.4	0.4883	0.4204	1.1615	0.2454	0.0441	NordNorge	Delusions narrow
0.001	0.0355	0.4319	0.0823	0.9344	0.0002	NordNorge	Delusions narrow
0.01	-0.1956	0.4250	-0.4602	0.6454	0.0064	NordNorge	Delusions narrow
0.05	-0.3646	0.5097	-0.7154	0.4744	0.0158	NordNorge	Delusions narrow
0.1	-0.3928	0.5260	-0.7467	0.4552	0.0176	NordNorge	Delusions narrow

0.2	-0.3020	0.5042	-0.5989	0.5492	0.0112	NordNorge	Delusions narrow
0.5	-0.3767	0.5130	-0.7344	0.4627	0.0170	NordNorge	Delusions narrow
1	-0.4188	0.5106	-0.8201	0.4122	0.0213	NordNorge	Delusions narrow
5e.8	0.0760	0.2329	0.3262	0.7443	0.0017	PADR	Delusions narrow
1e.5	0.2450	0.2496	0.9813	0.3264	0.0152	PADR	Delusions narrow
1e.4	-0.1482	0.2717	-0.5457	0.5853	0.0047	PADR	Delusions narrow
0.001	0.0498	0.2865	0.1737	0.8621	0.0005	PADR	Delusions narrow
0.01	0.3379	0.2721	1.2417	0.2144	0.0249	PADR	Delusions narrow
0.05	0.4238	0.2871	1.4758	0.1400	0.0355	PADR	Delusions narrow
0.1	0.4273	0.2827	1.5116	0.1306	0.0370	PADR	Delusions narrow
0.2	0.3956	0.2916	1.3565	0.1749	0.0297	PADR	Delusions narrow
0.5	0.4708	0.3134	1.5023	0.1330	0.0364	PADR	Delusions narrow
1	0.4614	0.3112	1.4828	0.1381	0.0354	PADR	Delusions narrow
5e.8	-0.0924	0.1390	-0.6651	0.5060	0.0022	REDIC	Delusions narrow
1e.5	0.0592	0.1386	0.4269	0.6694	0.0009	REDIC	Delusions narrow
1e.4	0.1325	0.1266	1.0464	0.2954	0.0055	REDIC	Delusions narrow
0.001	0.1773	0.1328	1.3352	0.1818	0.0090	REDIC	Delusions narrow
0.01	0.1113	0.1416	0.7856	0.4321	0.0031	REDIC	Delusions narrow
0.05	0.1300	0.1466	0.8865	0.3754	0.0039	REDIC	Delusions narrow
0.1	0.0515	0.1469	0.3509	0.7257	0.0006	REDIC	Delusions narrow
0.2	0.0848	0.1492	0.5685	0.5697	0.0016	REDIC	Delusions narrow
0.5	0.0733	0.1502	0.4879	0.6256	0.0012	REDIC	Delusions narrow
1	0.0644	0.1519	0.4238	0.6717	0.0009	REDIC	Delusions narrow
5e.8	0.1925	0.2952	0.6523	0.5142	0.0081	SAMAKS	Delusions narrow
1e.5	0.5499	0.3663	1.5011	0.1333	0.0458	SAMAKS	Delusions narrow
1e.4	0.3480	0.3508	0.9921	0.3211	0.0194	SAMAKS	Delusions narrow
0.001	0.3902	0.3911	0.9979	0.3183	0.0200	SAMAKS	Delusions narrow
0.01	0.2279	0.3143	0.7253	0.4683	0.0100	SAMAKS	Delusions narrow
0.05	0.0996	0.3672	0.2713	0.7862	0.0014	SAMAKS	Delusions narrow
0.1	-0.0639	0.3601	-0.1775	0.8591	0.0006	SAMAKS	Delusions narrow
0.2	-0.0694	0.3611	-0.1921	0.8477	0.0007	SAMAKS	Delusions narrow
0.5	-0.0682	0.3616	-0.1887	0.8503	0.0007	SAMAKS	Delusions narrow
1	-0.0540	0.3672	-0.1471	0.8830	0.0004	SAMAKS	Delusions narrow
5e.8	0.1246	0.1399	0.8907	0.3731	0.0040	IRCCS1	Delusions narrow
1e.5	-0.0240	0.1417	-0.1692	0.8656	0.0001	IRCCS1	Delusions narrow
1e.4	0.0025	0.1338	0.0185	0.9852	0.0000	IRCCS1	Delusions narrow
0.001	-0.0638	0.1388	-0.4594	0.6460	0.0011	IRCCS1	Delusions narrow
0.01	0.0405	0.1502	0.2698	0.7873	0.0004	IRCCS1	Delusions narrow
0.05	-0.0768	0.1509	-0.5091	0.6107	0.0013	IRCCS1	Delusions narrow
0.1	-0.0199	0.1494	-0.1332	0.8940	0.0001	IRCCS1	Delusions narrow
0.2	-0.0713	0.1534	-0.4646	0.6422	0.0011	IRCCS1	Delusions narrow
0.5	0.0018	0.1555	0.0115	0.9908	0.0000	IRCCS1	Delusions narrow
1	-0.0237	0.1560	-0.1522	0.8790	0.0001	IRCCS1	Delusions narrow
